# Imported Zika Virus in a European City: How to Prevent Local Transmission?

**DOI:** 10.3389/fmicb.2017.01319

**Published:** 2017-07-18

**Authors:** Joan-Pau Millet, Tomàs Montalvo, Ruben Bueno-Marí, Arancha Romero-Tamarit, Albert Prats-Uribe, Lidia Fernández, Esteve Camprubí, Lucía del Baño, Victor Peracho, Jordi Figuerola, Elena Sulleiro, Miguel J. Martínez, Joan A. Caylà, Zika Working Group in Barcelona, Dolores Álamo-Junquera

**Affiliations:** Servicio de Epidemiología. Agencia de Salud Pública de Barcelona; ICREA Movement Ecology Laboratory (CEAB-CSIC), Girona, Spain and CREAF (Center for Ecological Research and Forestry Applications); IRTA, Centre de Recerca en Sanitat Animal, CReSA, IRTA-UAB, Barcelona, Spain; ISGlobal, Barcelona Ctr. Int. Health Res. (CRESIB), Hospital Clínic-Universitat de Barcelona, Barcelona, Spain; Hospital Vall-d'Hebron-Drassanes, PROSICS, Barcelona, Spain; *Mosquito Alert* Community, Citizen scientists collaborating in the platform *Mosquito Alert*.; ^1^Servicio de Epidemiología, Agència de Salut Publica de Barcelona Barcelona, Spain; ^2^CIBER de Epidemiología y Salud Pública Barcelona, Spain; ^3^Servicio de Vigilancia y Control de Plagas Urbanas, Agencia de Salud Pública de Barcelona Barcelona, Spain; ^4^Laboratorios Lokímica, Departamento de Investigación y Desarrollo (I+D) Valencia, Spain; ^5^Unitat Docent de Medicina Preventiva i Salut Pública Parc Salut Mar-Universitat Pompeu Fabra-Agència de Salut Pública de Barcelona Barcelona, Spain; ^6^Estación Biológica de Doñana, Consejo Superior de Investigaciones Científicas Sevilla, Spain; ^7^Microbiology Department, Hospital Vall d' Hebron, PROSICS Barcelona, Universitat Autònoma de Barcelona Barcelona, Spain; ^8^Department of Microbiology, Hospital Clinic of Barcelona, Universitat de Barcelona Barcelona, Spain; ^9^ISGlobal, Barcelona Centre for International Health Research (CRESIB), Hospital Clinic of Barcelona, Universitat de Barcelona Barcelona, Spain

**Keywords:** arbovirus, epidemiology, global health, Guillain-Barré syndrome, microcephaly, public health, mosquito, Zika virus

## Abstract

**Background:** On February 1st 2016 the WHO declared the Zika Virus (ZIKV) infection a worldwide public health emergency because of its rapid expansion and severe complications, such as Guillain-Barré Syndrome or microcephaly in newborn. The huge amount of people traveling to endemic areas and the presence of *Aedes albopictus* in Barcelona increase the risk of autochtonous transmission. The objective of this study was to describe the first ZIKV cases diagnosed in our city and to analyze the surveillance, prevention, and control measures implemented to avoid autochthonous transmission.

**Methods:** An observational cross-sectional population-based study in Barcelona, Spain was performed.An analysis of the socio-demographic, epidemiological, clinical characteristics, and mosquito control activities of the ZIKV cases detected between January 1st and December 2016 was carried out using a specific ZIKV epidemiological survey of the Barcelona Public Health Agency.

**Results:** A total of 118 notifications of possible ZIKV infections were received, and 44 corresponded to confirmed cases in Barcelona residents.Amongst these, the median age was 35 years and 57% were women. All cases were imported, 48% were Spanish-born and 52% foreign-born. Dominican Republic was the most visited country amongst foreign-born patients and Nicaragua amongst Spanish-born. The most frequent symptoms were exanthema, fever, and arthralgia. Among the 24 diagnosed women, 6 (25%) were pregnant. There was one case of microcephaly outside Barcelona city. Entomological inspections were done at the homes of 19 cases (43.2% of the total) and in 34 (77.3%) public spaces. Vector activity was found in one case of the 44 confirmed cases, and 134 surveillance and vector control were carried out associated to imported ZIKV cases. In all cases prevention measures were recommended to avoid mosquito bites on infected cases.

**Conclusion:** Epidemiological and entomological surveillance are essential for the prevention of autochthonous transmission of arbovirosis that may have a great impact on Public Health.The good coordination between epidemiologists, entomologists, microbiologists, and clinicians is a priority in a touristic city with an intense relationship with endemic countries to minimize the risk of local transmission by competent vectors.

## Introduction

Arboviruses are a group of viruses transmitted through arthropods and many of them are capable of producing infection in humans. West Nile virus (WNV), Chikungunya (CHIKV), dengue (DENV), or Zika virus (ZIKV) are emerging arboviruses with a high potential of generating epidemic outbreaks (White et al., 2016). ZIKV was discovered in 1947 in Uganda by scientists that were performing a surveillance of the yellow fever virus in a forest called Zika (Hayes, 2009; Bulletin of the World Health Organization, 2016). This virus is transmitted by mosquitoes and *Aedes aegypti* is the main known vector (Ayres, 2016; Chouin-Carneiro et al., 2016). Recent infectivity studies in laboratory conditions revealed that *Aedes albopictus* is also susceptible to ZIKV virus infection, since the virus is replicated, disseminated and can reach to salivary glands. However, the efficiency of this infective process is lower in comparison with *A. aegypti*, which clearly shows the highest ZIKV vector competence among mosquitoes (Chouin-Carneiro et al., 2016; Di Luca et al., 2016; Jupille et al., 2016). Moreover, it is important to note that *A. albopictus* has been found infected also in wild populations in endemic areas as Gabon, in Central Africa (Grard et al., 2014).

The first cases of infection by ZIKV in humans were diagnosed in Uganda and Nigeria in 1952 (Macnamara, 1954). Throughout the second half of 20th century, the ZIKV had expanded to countries in Africa and Asia; India, Egypt, Malaysia, Mozambique, Nigeria, the Philippines, and Vietnam. Until 2007, only 16 cases in humans had been reported. It is at this time, when the ZIKV expands to the Yap Island, in the Pacific (Federal State of Micronesia), where the first great outbreak was reported (Duffy et al., 2009). Other large outbreaks were reported in the French Polynesia (2013–2014) and in Brazil in 2015, where the first ZIKV autochthonous case in Latin America was reported. This outbreak has been the origin of the Public Health crisis initiated in 2016. By the starts of February 2016, local transmission of ZIKV had been reported from more than 20 countries and territories in the Americas (Bulletin of the World Health Organization, 2016).

On February 1st 2016 the World Health Organization (WHO) declared the ZIKV epidemic a worldwide health emergency. This took place after the appearance of three epidemiological alerts related to outbreaks of congenital microcephaly and Guillan-Barré Syndrome (GBS) cases due to ZIKV in countries such as Brazil, France, USA, and El Salvador (U. S. Department of Health and Human Services, et al., 2016). Although a decline in ZIKV infections has been reported in some countries, or in some regions of countries, surveillance needs to remain high (WHO, 2016a).

Within Europe, Spain is one of the countries with high risk of autochthonous cases of ZIKV infection (WHO, 2016b), due to the presence of *A. albopictus* (Grard et al., 2014; Bueno, 2016) (commonly named “tiger mosquito”) in various regions and due to the great cultural, commercial, touristic, and migratory relationship with Latin America (Díaz-Menéndez et al., 2016). Consequently in April 2016 a National Plan of preparedness and response against DENV, CHIKV, and ZIKV was established. Its objective was to reduce the impact and the risk of establishment of these emerging diseases in Spain (MSSSI, 2016).

In Spain, the first imported case of ZIKV was detected in December 2015–January 2016 (Bachiller-Luque et al., 2016). In Barcelona, both imported ZIKV cases and *A. albopictus* (a competent vector for ZIKV) overlap in space and time. With the objective of controlling the imported ZIKV cases and prevent autochthonous cases, the public health services of the city were reorganized. This rearrangement was included within a more global Surveillance and Control of Arbovirosis Program that was already in place since 2014 that includes other emerging viruses transmitted by *A. albopictus* such as DENV, WNV, and CHIKV (Agència de Salut Pública de Catalunya, 2015; González et al., 2017).

The objectives of this paper were to describe the epidemiological and entomological surveillance for ZIKV cases in Barcelona (Spain) and to describe the improvements in the procedures to control ZIKV and other emerging arbovirosis to reduce the risk of an autochthonous outbreak.

## Methods

### Design

A descriptive observational cross sectional population-based study was performed in the city of Barcelona.

### Study period and population

The notified and confirmed ZIKV cases among residents in Barcelona city from January 1st to December 31st 2016 were studied. All cases were detected and followed by the Epidemiology Service of the Public Health Agency of Barcelona (PHAB) through the notifiable disease register. Entomological surveillance period starts on March and finishes on November because the low temperatures prevent vector activity. The cases that did not reside in Barcelona and those that were not confirmed by the laboratory were excluded from the analyses.

**Figure 1:**
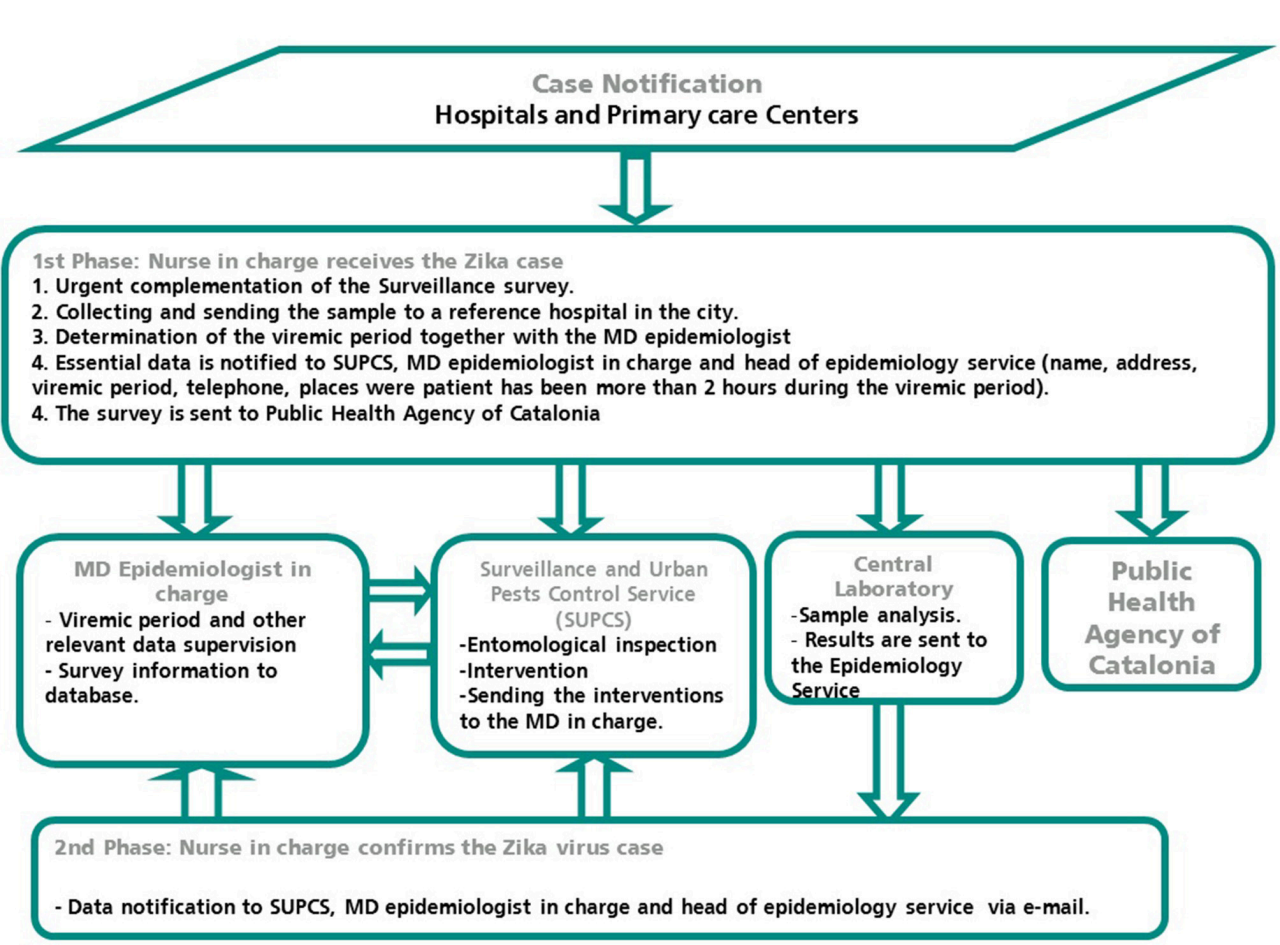
Coordination diagram from notification of a case of ZIKV infection to the intervention of the entomologist in the city of Barcelona.

ZIKV, CHIKV, and DENV are diseases of mandatory notification in Spain. In Barcelona an active epidemiological surveillance system is carried out based on a close communication among doctors, laboratories and the public health nurses from the Epidemiology Service. The circuit and procedures followed from the notification of a possible case until entomological inspections are listed in Figure 1.

### Definitions and case classification

The case definition used at the PHAB is described in the “National Plan for preparation and response against vector transmitted diseases” and in the “Protocol for surveillance and Control of mosquito transmitted arbovirosis in Catalonia” updated on 14 June 2016. The cases were classified according to clinical, epidemiological and laboratory criteria in four categories (Díaz-Menéndez et al., 2016; González et al., 2017).

1. *Probable case:* person that complies with the clinical criteria with or without epidemiological criteria and that complies with the laboratory criteria of probable case.
2. *Confirmed case:* person that complies with the clinical criteria with or without epidemiological criteria and that complies with the laboratory criteria of confirmed case.
3. *Imported case:* when the beginning of the symptoms occur until 15 days after abandoning a ZIKV epidemic area.
4. *Autochthonous case:* if there is no record/history of trip to endemic area within the last 15 days previous to the beginning of symptoms (Agència de Salut Pública de Catalunya, 2015).

The days of viremic phase in Barcelona since the arrival date were calculated for each patient. The viremic period was estimated from the natural history of the infection reported in the literature. For the viremic period, we assumed 7 days starting from the day of symptoms onset. However, we also simulate how many people would be viremic in Barcelona with different theoric values: 8 and 9 days of viremic period.

### Variables and source of information

The different epidemiological variables were collected from the epidemiological survey of Catalunya specific to ZIKV infection. Socio-demographic variables (age, sex, country of origin, neighborhood/district of residence), clinical variables (date of beginning of symptoms, viremic period, fever, asthenia, artharlgia, arthritis, conjuntival hyperemia, cephalea, no-purulent conjunctivitis, exanthema, myalgia, complications, hospitalization), diagnostic variables [date of diagnosis, laboratory results (IgG, IgM, PCR)], and epidemiological variables (way of transmission, pregnancy, and gestation week if affirmative, country or countries visited, dates of departure, and arrival to Barcelona, cause of trip, number of bites, place in which bites where received, mobility of the cases and if activities of prevention, confinement, or personal protection were given) were collected. The median time elapsed (in days) between the symptoms onset of patients and the first medical consultation, the notification and the laboratory confirmation, and the implementation of vector control activities were calculated.

The Surveillance and Urban Plague Control Service (SUPCS) at PHAB recorded different information during their entomological inspections related to the cases: places visited during the viremic period, vector activity at case residency, capture of adult vectors with BG traps, detection of mosquito breeding sites, and presence of virus in the vector.

### Surveillance procedures and vector control

In order to minimize the risk of ZIKV transmission through local mosquito bites during the viremic period, prevention measures where recommended by the Epidemiology Service. These measures included personal protection measures against mosquito bites (type of clothing, use of mosquito repellent, etc.), and house confinement during the viremic period and recommendations regarding safe sex.

Based on the collected information in the epidemiological survey, the SUPCS carried out an entomological inspection in at least two places: the public space around the case's place of residence and in the patient's own home. All the risk areas for the proliferation of mosquitoes were included in a Geographical Information System (GIS) to speed up the inspections. Gutters, fences, ornamental fountains and small artificial containers, within a buffer of 150 m in relation to the case's place of residence (habitual flying ratio of *A. albopictus* in the urban setting) were included in the GIS.

In our study, informative notes with recommendations to prevent the proliferation of larvae breeding sites in the home were distributed during the inspections of more vulnerable residential areas close to the imported case. The entomological inspections in private homes were analogous to the already described procedures for public areas. The areas were inspected for detecting larvae breeding spot sites and adult mosquitoes. It's worth mentioning that these activities are essential in the reduction of the risk of disease expansion because the contact between vectors and the infected host is likely to occur at home. The need to obtain an authorization from the patient to be able to carry out the inspection was an important limiting factor in comparison with the interventions in public spaces.

The collected entomological material was examined and identified and females were analyzed in pools for the detection of virus presence by RT-PCR. All the necessary steps for the evaluation of transmission risk were considered in the protocol, including vector activity (inside and around the patient's place of residence), and the efficacy of the control activities. The program monitoring the cases of imported arbovirosis is integrated within a general procedure that monitors and treats monthly the areas with recurrent proliferation of tiger mosquitoes (Bonnefoy et al., 2008; Montalvo et al., 2016).

### Statistical analysis

A descriptive study of qualitative and quantitative variables to characterize the study population was carried out. We computed the frequency distributions of the qualitative variables, and compared proportions using the χ^2^-test, or the two-tailed Fisher test when expected values were <5. Absolute frequencies were calculated for categorical variables. Social, demographic, epidemiologic and clinical variables were compared according to the country of origin of the infected person (Spanish born or foreign-born). The median and interquartilic range (IQR) for continuous variables was calculated and Spanish born and foreign born were compared using the U-Mann Whitney-test. A *p* < 0.05 was considered statistically significant.

### Ethical considerations

This study was approved by the Clinical Research Ethical Committee of Parc Salut Mar (IMAS). In order to guarantee data and registry confidentiality the regulation established by the Organic Law of Personal Data Protection of Spain 15/1999 and Royal Decree 994/1999 about security of computerized files that contain personal data was followed. All ethical principles for investigation in humans defined in the Declaration of Helsinki of 1964 revised and updated by the Worldwide Medical Association (Fortaleza, Brazil 2013) were followed.

## Results

### Epidemiological surveillance

**Figure 2:**
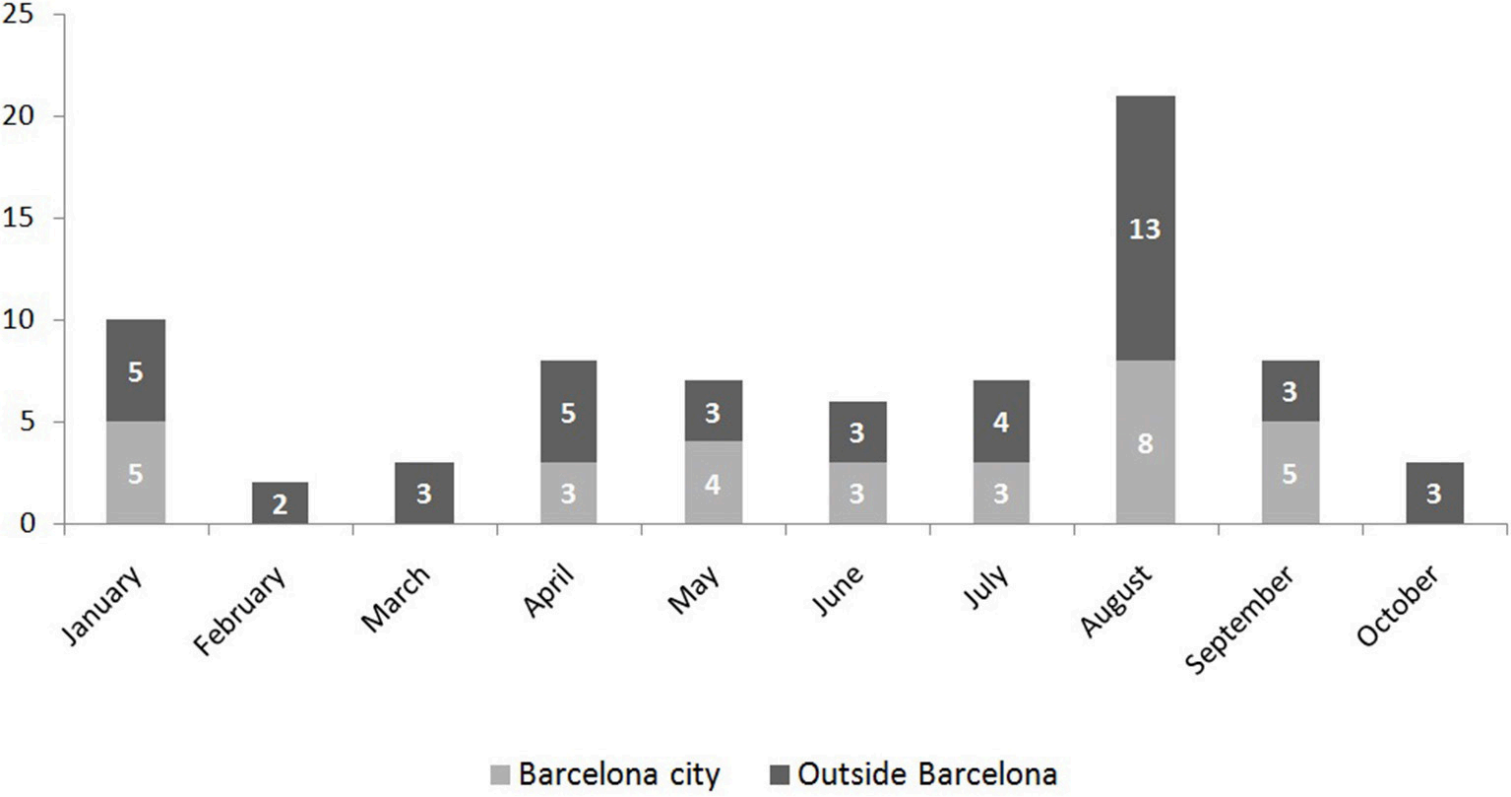
Monthly distribution according to place of residence (living in the city or outside the city of Barcelona) of the first 75 confirmed cases of Zika virus infection in Barcelona. Period: January–December 2016.

**Figure 3:**
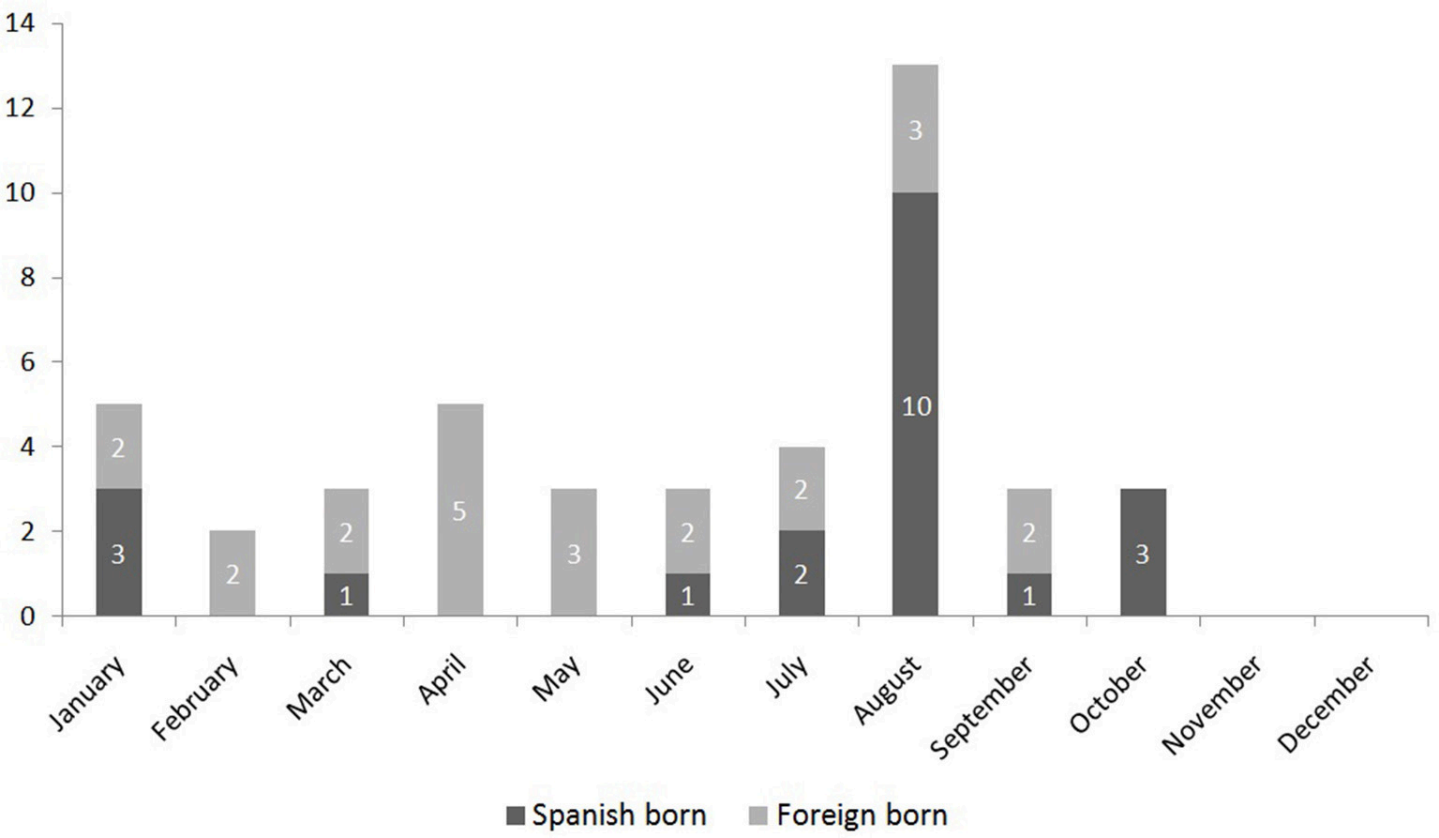
Monthly distribution of the 44 imported cases of Zika virus in Barcelona according to country of birth (Spanish born or foreign born). Period: January–December 2016.

During the study period 118 cases where notified, 75 of which (63.6%) were laboratory confirmed. Figure 2 shows the monthly distribution according to the place of residency (those who lived in the city or outside the city but diagnosed in Barcelona) of these 75 confirmed cases. Forty-four confirmed cases correspond to residents in the city, with an incidence of 2.74 cases per 100,000 inhabitants. The median age was 35 (standard deviation 11.8), 25 (57%) were women, and 19 (43%) men. They were all imported cases, 21 (48%) among Spanish-born population and 23 (52%) among immigrant population. No differences regarding age and sex were observed (*p* = 0.2 and 0.57, respectively). The number of cases peaked in August, when most of the infections occurred among Spanish-born individuals (Figure 3).

When comparing the characteristics of the 44 confirmed cases between Spanish-born and foreign-born, it stands out that the most frequently visited countries where the Dominican Republic and Venezuela amongst the foreign-born and Nicaragua, Colombia, Mexico, and Vietnam amongst the Spanish-born. Fifty-two percent of the cases didn't report being bitten by mosquitoes after arrival to Barcelona. The most frequent clinical symptoms were rash (86%), fever (61%), and arthralgia (59%). No significant differences were found between Spanish-born and foreign-born in relation to the clinical symptoms, visited country or detected bites (Table 1).

**Table 1:** Comparison of the descriptive characteristics between Spanish born and foreign born for the 44 confirmed cases of Zika virus infection in the city of Barcelona. January–November 2016.

	**Spanish-born N (%)**		**Foreign-born N (%)**		**Total N (%)**	
	21	48%	23	52%	44	
**AGE (MEDIAN, SD)**	33.5	10.7	36.5	12.7	35	11.8
**SEX**						
Male	10	48%	9	39%	19	43%
Female	11	52%	14	61%	25	57%
**HOSPITALIZATION**						
Yes	0	0%	0	0%	0	0%
No	21	100%	23	100%	44	100%
**CLINICAL SYMPTOMS**						
Fever	14	67%	13	57%	27	61%
Arthralgia	11	52%	15	65%	26	59%
Rash	17	81%	21	91%	38	86%
Myalgia	6	29%	7	30%	13	30%
Cephalea	9	43%	9	39%	18	41%
Any other symptom	15	71%	12	52%	27	61%
**COMPLICATIONS**	0	0%	0	0%	0	0%
**VISITED COUNTRY**						
Dominican Republic	2	10%	6	26%	8	18%
Nicaragua	6	29%	2	9%	8	18%
Colombia	3	14%	2	9%	5	11%
Mexico	3	14%	1	4%	4	9%
Venezuela	0	0%	4	17%	4	9%
Vietnam	3	14%	0	0%	3	7%
Honduras	1	5%	1	4%	2	5%
Bolivia	0	0%	2	9%	2	5%
Others	3	14%	5	22%	8	18%
**DETECTED BITES**						
Yes	10	48%	11	48%	21	48%
No	11	52%	12	52%	23	52%
**PREGNANCY**	2	10%	2	14%	4	16%
**DAYS OF RISK IN BARCELONA (VIREMIC PERIOD)**						
0 days	10	48%	3	13%	13	30%
1–7 days	5	24%	6	26%	11	25%
From 8 to 9 days	6	29%	14	61%	20	45%
**PREVENTIVE RECOMMENDATIONS**						
Yes	21	100%	23	100%	44	100%
No	0	0%	0	0%	0	0%

**Figure 4:**
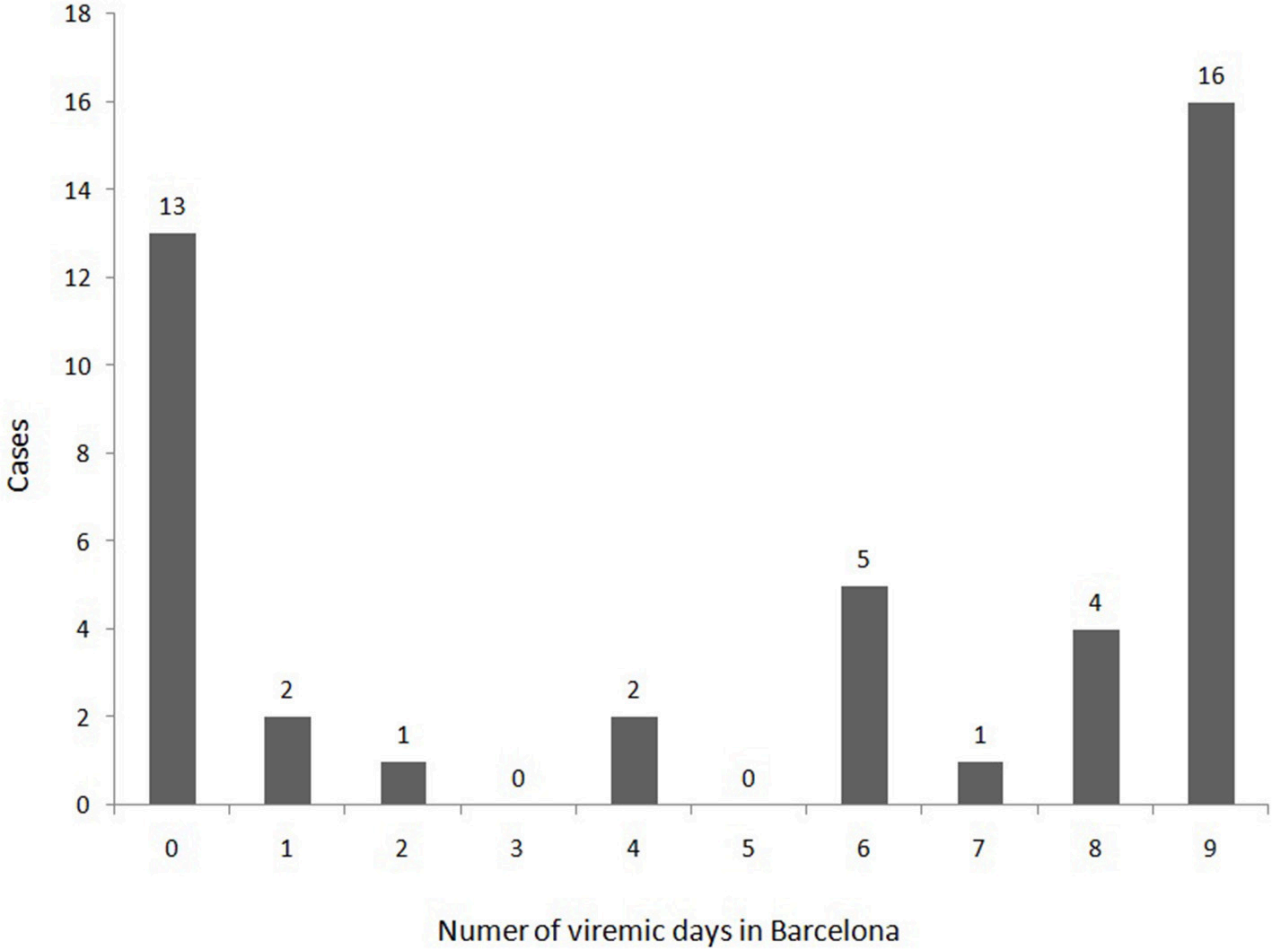
Distribution of the first 44 confirmed cases of Zika virus infection according to the viremic days spent in the city of Barcelona.

A total of 31 cases (70.5%) were arrived in Barcelona during the viremic period and 13 (30%) arrived during the incubation phase and became viremic in the city (Figure 4). The proportion of viremic immigrants for 8–9 days in Barcelona was higher than for the Spanish born (Table 1) (61% vs. 29%), *p* = 0.034.

The median time, for Spanish born, elapsed between the symptoms onset and: (a) the first medical consultation date was 4 days (IQR 2–5); (b) the notification date was 8 days (IQR 7–14); (c) the laboratory confirmation date was 9 days (IQR 6–12), and (d) the implementation date of vector control activities was 9 days (IQR 7–14). The median time in days for foreign born was 4 (IQR 2–11), 11 (IQR 8–27), 20 (IQR 13–25), and 13 (IQR 11–28), respectively. No statistically significant differences were observed in these elapsed times between Spanish-born and foreign-born patients except for the lab confirmation days (*p* = 0.04).

Regarding the consequences of the ZIKV infection, no mortality was observed. Regarding morbidity, no GBS was detected. Among the 24 diagnosed women, 6 (25%) were pregnant. There was one case of microcephaly notified to PHAB but the pregnant woman lived outside Barcelona city.

### Vector control

**Figure 5:**
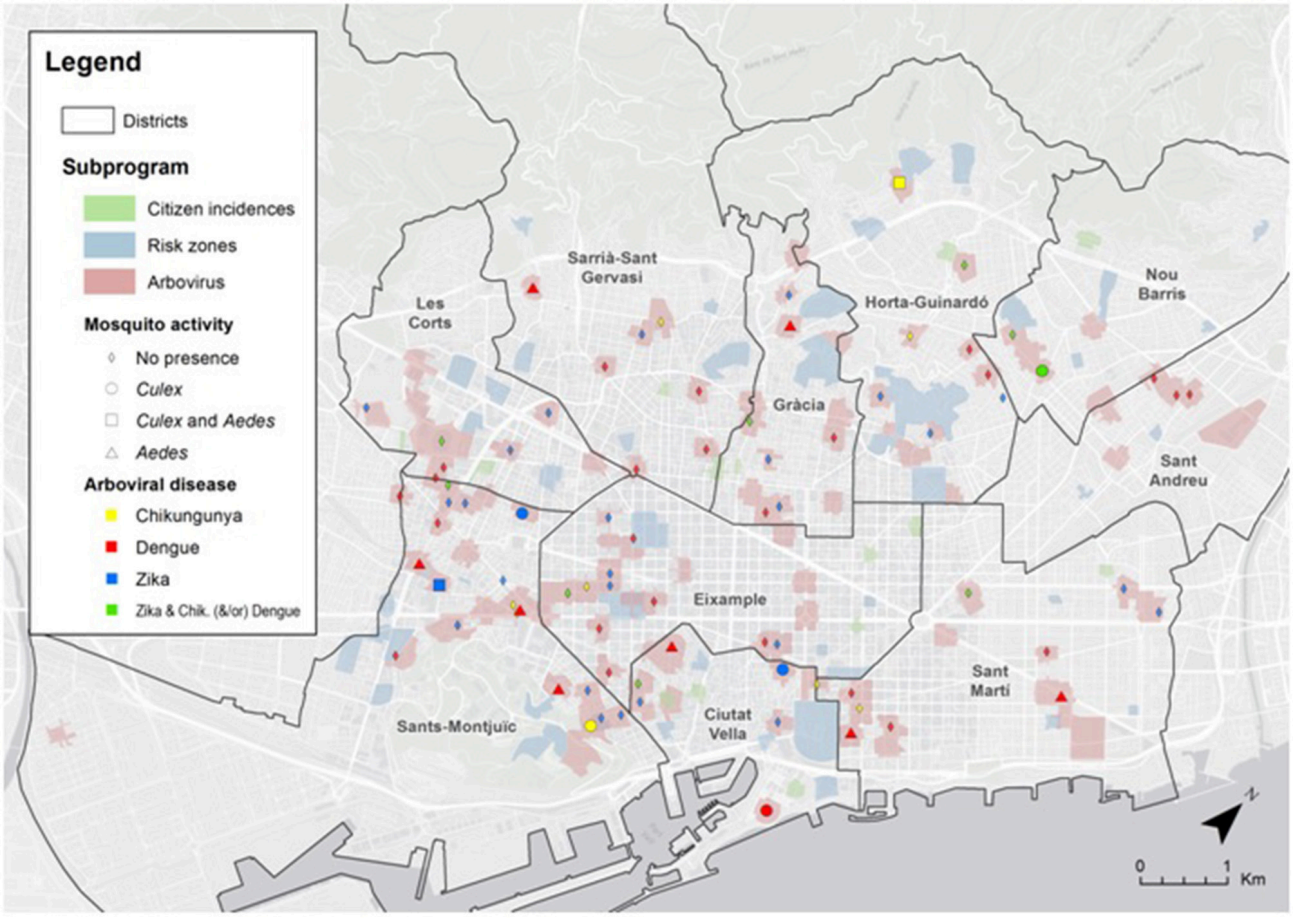
Map of the city of Barcelona with the spatial location of the different arbovirosis cases and the results of the entomological inspections. Risk zones: monthly surveillance and control vector zones. Citizen incidences: Notices regarding mosquito problems that citizens have indicated during the study period.

According to the Surveillance protocol of arbovirosis in Catalonia, 34 entomological inspections related to ZIKV were done between 1st April and 15th November. Nine of the 34 cases had high mobility during the viremic period visiting different areas of the city, increasing their exposure to tiger mosquito bites. Only 19 (43.2%) homes could be inspected, since the persons could not be contacted or refused the inspection in the other 15 cases. The activity of *A. albopictus* was low, and was only detected in the public neighborhood of one of the cases (Figure 5).

**Figure 6:**
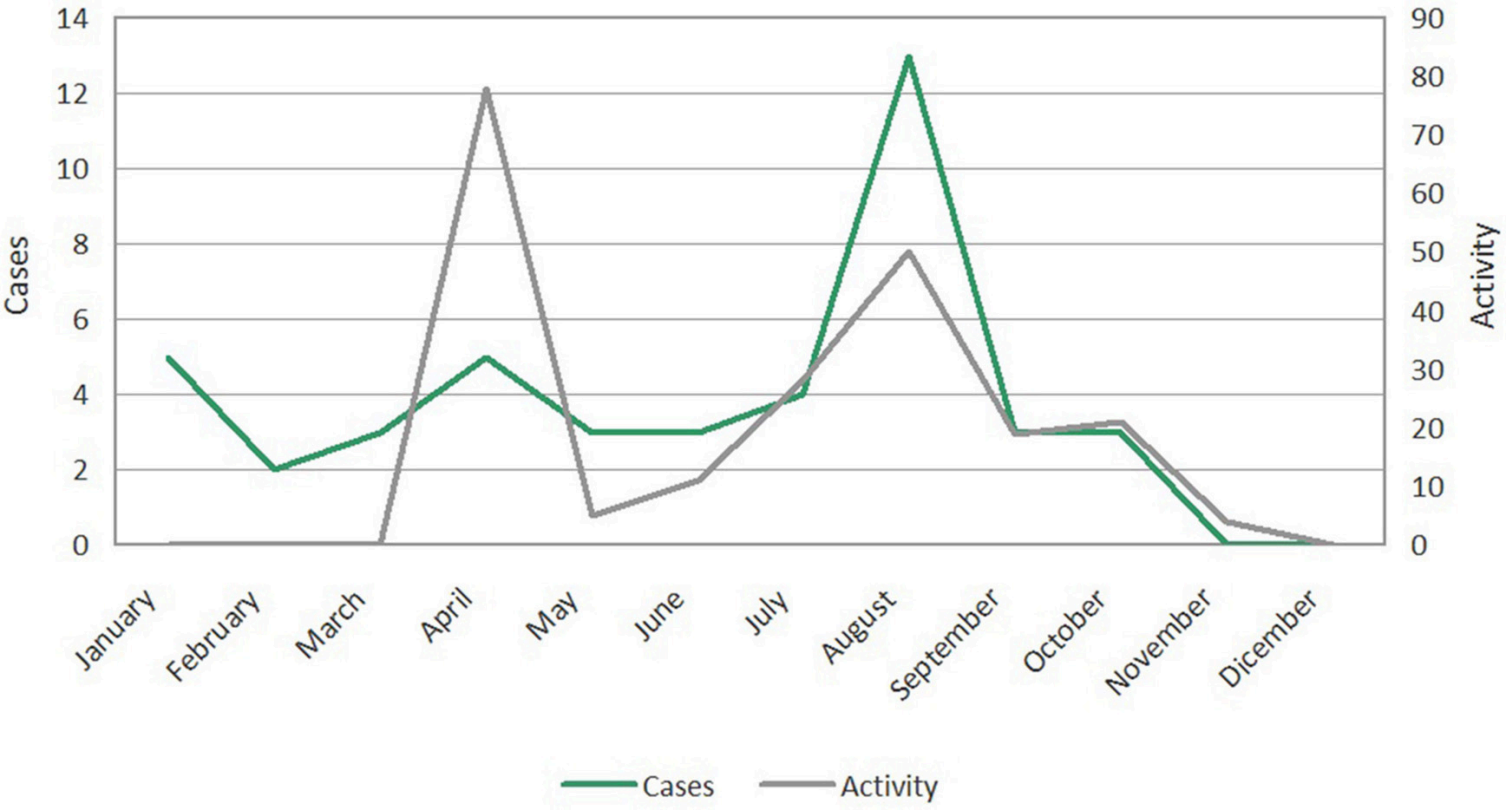
Distribution of the Zika virus and *Aedes albopictus* vector activity (number of larval hatcheries detected on the public road) by month in the city of Barcelona.

There was a seasonal overlap between vector activity, and the arrival of imported ZIKV cases with viremia (Figure 6). April, August, and September, are amongst the months of higher overlap and consequently higher risk of local transmission of ZIKV.

To minimize the risk of transmission as much as possible, 122 vector control interventions took place in public spaces and 12 in places of residence of ZIKV cases. Most of the interventions were larvicide treatments in gutters and modifications of elements with risk of hosting mosquito larvae, in most cases in private property. In those cases in which tiger mosquito activity was detected or its presence was suspected, we used BG Sentinel traps for capturing mosquito females. Twelve traps were set, four in public spaces, and eight in private property. Only one pool of mosquitoes was finally analyzed for ZIKV detection and the result of the molecular analysis was negative.

## Discussion

The first year of epidemiological surveillance for ZIKV in the city of Barcelona has allowed us to know very well the profile of ZIKV imported cases. The wide presence of a vector that can transmit the virus (*A. albopictus*) and the population movements increases the risk of introduction of emerging arbovirusis like ZIKV. This fact requires surveillance of the imported cases and tasks for vector control. This makes a good coordination between all the different actors, like epidemiologists, clinicians, entomologists, and microbiologists essential in order to avoid ZIKV from becoming a public health problem that would also affect the tourism in the city.

The imported ZIKV cases profile was that of a traveler that after visiting endemic areas for ZIKV in Latin America, presents clinical symptoms such as fever, rash and arthralgia. No differences in sex, age, clinical symptoms, detected bites, or visited country were observed among Spanish and foreign-born patients. Regarding the visited country, the predominant group was originally from the Dominican Republic, Venezuela, and Nicaragua or Colombia. Other cities in Spain have also reported a high number of cases from Latin American countries. This is due to the mobility of people between Latin America and Spain that takes place for different reasons such as business, cooperation, tourism or visits to friends and relatives. It also responds to the high number of immigrants living in Spain especially since year 2000, and that occasionally travel to their countries of origin (López De Lera, 1995; Díaz-Menéndez et al., 2016). In many of these countries ZIKV is endemic and its expansion is favored by the wide presence and abundance of *A. aegypti*, main vector for ZIKV and other arbovirosis.

### Viremia

Over 70% of the diagnosed ZIKV cases in our city where viremic (PCR positive on serum) when they sought medical attention and over 36% (16/44) arrived in the city during incubation phase and spend all the viremic phase in our city. Usually tourists spend less time at their destinations than foreign born which also explains that Spanish-born patients more often present viremic phase after arriving. For the foreign-born, the reason for traveling is to visit friends and relatives and therefore normally they stay with their family and stay for a longer period of time. The cases that started viremic phase after arriving to Spain represented the highest risk for local transmission because of the longest duration of exposure to local vectors while still viremic. Additionally, an important percentage of patients reported mosquito bites after arrival, in many cases overlapping with the viremic period.

The incubation period can vary between 2 and 14 days after infection and the viremic period can be 3–5 days long or longer (Falcao et al., 2016). Nevertheless, the viremic period for ZIKV has not been clearly established, especially in some vulnerable populations such as pregnant women (Suy et al., 2016). The viremic period in our city is estimated from the natural history of the infection, 7 days starting from the day of symptoms onset, not performing a repeated blood test to check the presence of the virus. Recently the CDC stated that the ZIKV viremic period should be extended to 7 days, information that is of great relevance in vector control and autochthonous transmission (Agència de Salut Pública de Catalunya, 2015). According to Figure 4 it can be deduced that if the viremic phase was considered to be only 5 days, the total days for all cases in viremic period would be 116 days. However, if the viremic phase is considered to be 9 days, the total days of viremic period are 252 days, almost twice. Therefore, we believe that while the accurate length of the viremic phase is not yet established, the best option to maximize effects of epidemiological surveillance and entomological precautions is to consider a viremic phase of 9 days (Alejo-Cancho et al., 2016).

The duration of the viremic period has also important implications for the establishment of prevention measures. Entomologic inspections and tasks should occur as soon as a suspected case seeks medical advice, in order to reduce the risk of transmission during the viremic phase. Delays in the flow of information between medical and entomological staff may dramatically reduce the effectiveness in control, making them ineffective if the information reaches the entomological staff when the viraemic period has finished.

It is important to highlight that 9 of 34 confirmed cases, in spite of the recommendations given by public health services or the clinic, experienced great mobility during the viremic period. The fact that the clinical symptoms are frequently mild probably had an influence on this. These aspects together with the fact that most of the imported cases overlapped with periods of vector activity increased the risk of possible transmission. It is therefore very important to recommend to patients a reduction in their mobility during this period (Marrama-Rakotoarivony and Zeller, 2012).

### Zika virus consequences

To this date, nor deaths nor cases of SGB related to ZIKV have been reported in Barcelona. However, in a retrospective case-control study that took place after the ZIKV outbreak in French Polynesia (2013–2014), 42 cases of SGB where identified which were also positive for DENV virus and ZIKV. Recently there has been an increase in the SGB incidence in countries like Brazil, Colombia, El Salvador, and Suriname, but the exact cause is still unknown.

Unfortunately, we found one microcephaly case out of 6 babies born from ZIKV infected mothers. We had a high portion of pregnant woman due to the active screening of pregnant woman that is being performed in Catalonia. Not only the doctors but also pregnant woman that travel to a ZIKV endemic area are aware about the risks of microcephaly in newborn and look for medical advice and follow up when they are back home. This ongoing protocol in Catalonia perform not only an screening and follow up of all pregnant woman exposed to the risk of ZIKV but also a long term follow-up of all newborn in order to monitor the development of neurological abnormalities (Bocanegra et al., 2016). A prospective study in Brazil amongst pregnant women infected by ZIKV showed that 29% of the fetus suffered some type of abnormality during pregnancy, including microcephaly and intrauterine restricted development. Fetal abnormalities were detected by Doppler ultrasonography in 12 of the 42 (29%) ZIKV positive women (Brasil et al., 2016; Mysorekar and Diamond, 2016; Reynolds et al., 2017). In light of this scenario, we need to rapidly evaluate the risk to child development by this emerging disease that is spreading quickly in many territories.

In the context of epidemiological surveillance it is important not to forget that ZIKV could be a sexual transmitted disease (STD). In the latest WHO update, 12 countries have reported STD of ZIKV, not only from man to woman, but also from woman to man, man to man (Deckard et al., 2016) and also could be transmitted by oral sex (World Health Organization, 2016). The exact duration of ZIKV in semen still remains unknown. In a recent study, ZIKV RNA was detected in semen 62 days after the initiation of the symptoms (D'Ortenzo et al., 2016), even though the longest period in which ZIKV has been detected in semen is 188 days (Althaus and Low, 2016; Paz-Bailey et al., 2017). Other routes of transmission include transmission through blood (Barjas-Castro et al., 2016).

### Reorganization of the public health services

After the diagnosis of the first imported cases of arbovirosis in our city, it was necessary to face up to the risk the presence of *A. albopictus* represented to public health. For these reason, an epidemiologist and an entomologist were incorporated in the team for surveillance and control of arbovirosis.

As a result, a protocol for the surveillance and control of mosquito-borne arboviruses was established in Catalonia in 2014. It was written by an inter-institutional commission comprised of different experts (clinicians, microbiologists, entomologists, epidemiologists, etc., Agència de Salut Pública de Catalunya, 2015). The importance of surveillance and early detection was key therefore an interdisciplinary group formed by members of the Epidemiology Service and the Vector Control Service of the PHAB was created. Public health nurses were trained to fully know the arboviruses and to adequately complete the case reporting surveys. The structure (Figure 1) was also organized in coordination with the doctors of the hospitals specialized in Travel Medicine. This changes improved communication systems, and accelerated the responses to risk situations that occurred with the arrival of viremic patients. A fundamental aspect was the communication and knowledge of the protocol by all the involved agents. For this reason, PHAB organized training sessions for physicians were held in the main hospitals. The aim was to increase awareness of the importance of each agent involved in the process (Bonnefoy et al., 2008; Bueno, 2016). The information was disseminated through the commission of arbovirosis of Catalonia and through TV programs and news reports and at a round table that was organized at the 6th Emerging Diseases Congress in Barcelona (Camprubí, 2016).

In this first year of experience in ZIKV surveillance and control, the delays observed among time elapsed between the symptoms onset and first medical consultation, public health notification, laboratory confirmation, and implementation of vector control activities, were remarkable. The first problem was that the diagnostic delay in the first medical consultation and therefore the notification delay to the public health system. Therefore, the vector control activities were also initiated later, when patient was no longer viremic. So, from public health it is essential to educate patients to consult soon and also that doctors notify all suspected cases of ZIKV quickly.

### Vector control

To this date no autochthonous ZIKV case has been detected in Barcelona. However, they could have been occurred since some cases may goes under detected as happened in Croatia for DENV infection (Kurolt et al., 2013). There is a clear overlap of the peaks of imported cases and the vector's activity (Figure 6). The analysis of the phenology in the vector appearance using a weighting of the risk factors, allows us to identify the months of April, August, and September (in general the months with highest temperatures) as the most favorable for the mosquito species (Figure 3). In any case, the phenology of *A. albopictus* matches the predictive models and the observations that have been carried out recently in different Mediterranean cities (Tran et al., 2013; Bueno, 2016).

Local transmission of ZIKV has been observed recently in temperate zones of USA such as Texas and Florida. Among the 1,325 reported cases in 2016, 262 were due to local transmission, and 224 were in pregnant woman (16.9%). After governmental efforts on vector control activities, no new ZIKV cases have been detected by local transmission in Florida. The example of Florida together with Barcelona, underlines the importance of surveillance and vector control activities and close monitoring to prevent ZIKV circulation (Department of Health Daily Zika Update, 2017).

In light of this situation, efforts to intensify traveler's advice, preventive measures and individual protection measures must be maximized. We believe in paying special attention in providing resources to reduce mobility of the cases that are in viremic period. This would help reduce contact with the vector and decrease possible risk of transmission. A possible solution would be to make home confinement obligatory. We are aware that adherence to this recommendation could be low. In the case of people that are working, an obligatory medical leave could be provided in order to reduce mobility. Another area in which improvement could be made is in the inspection of private properties. These are confined areas where there is a great overlap between vector and viremic patient. In our case only 19 out of 34 homes could be inspected, leaving a significant part of the risk assessment without completion.

In Barcelona, when vector activity was detected, either in larval phase or in adult phase, larvicide or adulticide control actions took place within the framework of the basic criteria of Plague Integrated Control (Bonnefoy et al., 2008). Adulticide spraying can pose several environmental problems especially in urban environments, since they are of large spectrum interventions that can affect non-target organisms. However, although the effect of adulticide treatments is well known to be short-lived, in certain epidemiological contexts a well technified adulticide application is needed to reduce the mosquito population rapidly (Caputo et al., 2016). Consequently, it is a particularly interesting strategy in the framework of imported cases of arbovirus in a concrete place where the vectors are present in high densities. In any case, it is well known that a long temporal lapse between outbreak initiation and the start of control tasks reduces effectiveness of adulticides applications significatively in terms of cost-benefits (Burattini et al., 2008). Therefore, the best approach is to supply reactive adulticides by preventive larvicides as a basis of the control programme, since the treatment of potential breeding sites with larvicides has a proven role in the reduction of adult population at local scale in urban environments (Sochacki et al., 2016).

The activity of vectors detected in the entomological inspections of the ZIKV viremic cases in homes was low. However, additional inspections related to DENV cases detected high activity for five cases during 2016. This fact highlights the need for coordination in the protocols for surveillance and control of any arbovirosis in order to reduce the risk of transmission (Lucientes-Curdi et al., 2014). Similar protocols have been implemented in other countries such as France or Italy (Zammarchi et al., 2015; Maria et al., 2016) and recently have also been approved at a national level in Spain (MSSSI, 2016). It is important that these programs for surveillance and control integrate epidemiological issues (with an appropriate identification and follow up of cases and adequate prevention measures) as well as vector issues (vector presence and quantification) (Bonnefoy et al., 2008; Bueno, 2016).

The use of new technology can help improve the systems and knowledge of the territory (mosquito proliferation areas and detection of new competent vectors for transmission of diseases). Applications such as *Mosquito Alert* (http://www.mosquitoalert.com) can help improve the early detection system and are of great help in managing this public health problem (Center for Ecological Research and Forestry Applications. Oltra et al., 2016). *Mosquito Alert* collaborated in locating unidentified breeding sites in private property near confirmed cases of ZIKV.

*A. albopictus* may not be the only competent vector of ZIKV in Barcelona. Different studies have suggested the possible role of Culex sp. as a ZIKV vector due to the occasional detection of the virus in wild populations (Huang et al., 2016). However, viral infection experiments in the laboratory have not confirmed Culex as a competent vector (Amraoui et al., 2016; Boccolini et al., 2016; Huang et al., 2016).

### Risk of local transmission and preventive measures

Autochthonous transmission of ZIKV can potentially occur in the Mediterranean countries since imported cases and competent vectors are present. Therefore, it is essential to establish active case surveillance and prevention protocols to avoid local transmission. As previously stated, the most effective prevention measure to avoid local transmission is vector control. Additionally, it is important that healthcare professionals are informed of the potential risk of ZIKV cases, since this will improve early case detection, surveillance procedures and transmission control (Leona, 2016; Red Nacional de Vigilanca Epidemiológica, 2016). Nevertheless, since a low percent of ZIKV are symptomatic, the majority of viremic travelers returning from endemic zones could be unidentified, and therefore possible source of autochthonous outbreak. In this sense, reducing mosquito populations in the city may significantly reduce the risk of autochthonous outbreak because limiting vector control activities to the neighborhood of symptomatic cases, may have limited impact if a significant fraction of ZIKV infections are asymptomatic during the viraemic phase.

In relation to the preventive measures, at an individual level, the imperative recommendation is that all people that travel to endemic areas should take precautions against mosquito bites (use of repellents, mosquito nets, long sleeve clothes) and on arrival should visit a medical center if any symptom develops (Agència de Salut Pública de Catalunya, 2015; Leona, 2016; MSSSI, 2016). At a community level, taking into consideration that in the last few years migratory mobility and the increase of individual's mobility has produced the introduction of emerging diseases such as many arbovirosis, the recommendations would be based on reducing the areas suitable for the reproduction of *Aedes* mosquitoes in around human inhabited areas. These measures would be based on the elimination or protection of small containers that can accumulate water, and the application of measures of control in those water containers that cannot be protected or eliminated. A frequent inspection of these spaces to avoid any mosquito breeding site would be necessary. Individual protection measures should be taken in order to avoid bites, such as the use of long sleeves and long pants, and the use of repellents.

Pregnant women are a priority. The early detection of the cases and effects on fetus and newborns is essential. In pregnant women, the diagnosis has to be confirmed and there has to be a follow up of the pregnancy and the fetus. Men arriving from areas with local ZIKV transmission should have safe sex during a minimum period of 6 months. Women arrived from endemic areas should have safe sex during 8 weeks (Red Nacional de Vigilanca Epidemiológica, 2016). The pregnant women who have traveled to areas with local ZIKV transmission or that have presented symptoms after the trip should communicate this information during pre-natal visits. The time that a woman has to wait to get pregnant after arriving from an affected area is 8 weeks from the time of arrival or since the time of diagnosis (CDC, 2016; WHO, 2016c).

All these preventive measures together with a good management of imported ZIKV cases and coordination among epidemiologists, clinicians, entomologists, and microbiologists are essential to prevent local transmission and therefore to limit the extension of this emerging disease. The arbovirus surveillance program in Barcelona is an example of the need of a multidisciplinary approach in order to reduce the risk of introduction of the different arbovirosis, amongst them, ZIKV. The coordination between public health and pest control agencies has contributed to the reduction of the risk of autochthonous transmission.

## Author contributions

JM, TM, RB, EC, and JC conceived and designed the work, write the first draft, revised critically and approved the final version. AR, AP analyzed and participated in the interpretation of the data, revised critically and approved the final version. AR, AP, LF, LD, VP, MM, ES, and JF participated in the acquisition and interpretation of the data, revised critically and approved the final version. Zika Working Group in Barcelona participated in the acquisition and interpretation of the data, revised critically and approved the final draft. All authors agree to be accountable for all aspects of the work.

### Conflict of interest statement

The authors declare that the research was conducted in the absence of any commercial or financial relationships that could be construed as a potential conflict of interest.

## Abbreviations

CHIKV: Chikugunya
DENV: Dengue
GBS: Guillain-Barré Syndrome
PHAB: Public Health Agency of Barcelona
STD: Sexual Transmitted Disease
SUPCS: Surveillance and Urban Plague Control Service
WHO: World Health Organization
ZIKV: Zika Virus.
